# 1-(3-Chloro­phen­yl)-4,4,6-trimethyl-3,4-dihydro­pyrimidine-2(1*H*)-thione

**DOI:** 10.1107/S1600536810054292

**Published:** 2011-01-08

**Authors:** Bohari M. Yamin, Halima Farag Salem

**Affiliations:** a School of Chemical Sciences and Food Technology, Universiti Kebangsaan Malaysia, UKM 43600 Bangi Selangor, Malaysia

## Abstract

In the title compound, C_13_H_15_ClN_2_S, the dihydro­pyrimidine ring is essentially planar, with a maximum deviation from the least-squares plane of 0.122 (3) Å for the unsubstitued olefinic C atom. The dihedral angle between the dihydro­pyrimidine and benzene rings is 86.62 (13)°. The crystal structure is stabilized by inter­molecular N—H⋯S hydrogen bonds, which form centrosymmetric dimers arranged along the *c* axis.

## Related literature

For related structures, see: Yamin *et al.* (2005 ▶); Ismail *et al.* (2007 ▶); Saeed & Bolte, (2010 ▶). For the biological activity of dihydro­pyrimidinone/thione derivatives, see: Alam *et al.* (2005 ▶); Kappe (2000 ▶); Sriram *et al.* (2006 ▶); Leite *et al.* (2006 ▶). For graph-set theory, see: Etter *et al.* (1990 ▶); Bernstein *et al.* (1995 ▶).
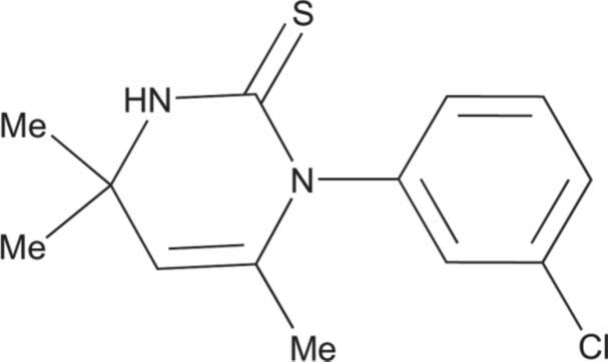


## Experimental

### 

#### Crystal data

C_13_H_15_ClN_2_S
*M* *_r_* = 266.78
Monoclinic, 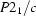

*a* = 8.398 (2) Å
*b* = 14.930 (4) Å
*c* = 11.468 (3) Å
β = 103.909 (4)°
*V* = 1395.7 (6) Å^3^
*Z* = 4
Mo *K*α radiation
μ = 0.40 mm^−1^
*T* = 298 K
0.40 × 0.19 × 0.17 mm

#### Data collection

Bruker SMART APEX CCD area-detector diffractometer
Absorption correction: multi-scan (*SADABS*; Bruker, 2000 ▶) *T*_min_ = 0.855, *T*_max_ = 0.934
8215 measured reflections
2598 independent reflections
2212 reflections with *I* > 2/s(*I*)
*R*_int_ = 0.021

#### Refinement

*R*[*F* ^2^ > 2σ(*F* ^2^)] = 0.048
*wR*(*F* ^2^) = 0.133
*S* = 1.10
2598 reflections
157 parameters
H-atom parameters constrained
Δρ_max_ = 0.29 e Å^−3^
Δρ_min_ = −0.14 e Å^−3^

### 

Data collection: *SMART* (Bruker, 2000 ▶); cell refinement: *SAINT* (Bruker, 2000 ▶); data reduction: *SAINT*; program(s) used to solve structure: *SHELXS97* (Sheldrick, 2008 ▶); program(s) used to refine structure: *SHELXL97* (Sheldrick, 2008 ▶); molecular graphics: *ORTEPIII* (Burnett & Johnson, 1996 ▶), *ORTEP-3 for Windows* (Farrugia, 1997 ▶) and *PLATON* (Spek, 2009 ▶); software used to prepare material for publication: *SHELXL97*, *PARST* (Nardelli, 1995 ▶) and *PLATON*.

## Supplementary Material

Crystal structure: contains datablocks global, I. DOI: 10.1107/S1600536810054292/dn2640sup1.cif

Structure factors: contains datablocks I. DOI: 10.1107/S1600536810054292/dn2640Isup2.hkl

Additional supplementary materials:  crystallographic information; 3D view; checkCIF report

## Figures and Tables

**Table 1:** Hydrogen-bond geometry (Å, °)

*D*—H⋯*A*	*D*—H	H⋯*A*	*D*⋯*A*	*D*—H⋯*A*
N1—H1*A*⋯S1^i^	0.85	2.58	3.404 (2)	162

Symmetry code: (i) 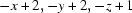.

**Fig. 1.**
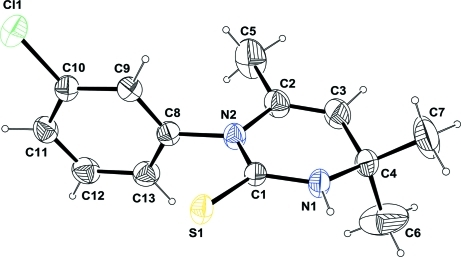
Molecular view of (I) with the atom-labelling scheme. Displacement ellipsoids are drawn at the 30% probability level. H atoms are represented as small spheres of arbitrary radii.

**Fig. 2.**
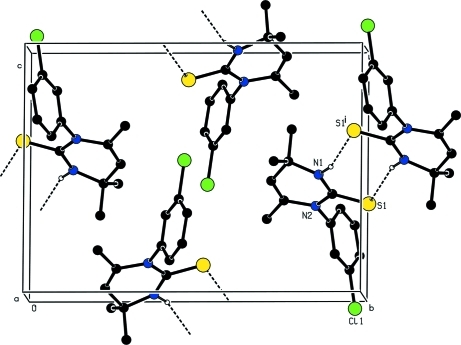
The molecular packing of (1) viewed down the a axis. H atoms not involved in hydrogen bondings have been omitted for clarity. H bonds are represented as dashed lines. [Symmetry code: (i) -x+2, -y+2, -z+1]

## supplementary crystallographic information

### Comment

The dihydropyrimidinone/thione derivatives are medicinally important due to their therapeutic and pharmacological properties (Kappe, 2000; Alam *et al.*,2005; Sriram *et al.*, 2006; Leite *et al.*, 2006)).

The title compound (I) is a *meta* isomer of the previously reported 1-(4-Chlorophenyl)-4,4,6-trimethyl-3,4-dihydropyrimidine-2(1 H) -thione (Saeed & Bolte,2010). The dihydropyrimidine N1/C1/N2/C2/C3/C4 ring is essentially planar with maximum deviation of 0.122 (3) Å for the unsubstituted olefinic carbon C3 atom compare to that in the *para* isomer where the C4 atom bearing the two methyl substituents deviated by 0.44 (2)%A from the other five almost coplanar atoms (Saeed & Bolte,2010). The dihedral angle between the dihydropyrimidine and benzene ring is 86.62 (13)° (Fig. 1), smaller than that in the *para* isomer of 89.59 (5) Å. The bond lengths and bond angles agree with closely related structures (Ismail *et al.*, 2007; Yamin *et al.*, 2005).

The molecular packing is also characterized by centrosymmetric dimers connected by the N—H..S intermolecular hydrogen bond forming a R_2_^2^(8) ring (Etter *et al.*, 1990, Bernstein *et al.*, 1995) and are arranged parallel to the *c* axis (Table 1, Fig 2).

### Experimental

The title compound was prepared by the reaction of thiocynic acid (5.4 mmol) and 3-chloroanaline (5.4 mmol) in acetone. The reaction mixture was stirred for 2–3 h. Then the clear was left for slow evaporation at room temperature. Colourless crystals of 1-(3-Chlorophenyl)-4,4,6-trimethyl -3,4-dihydropyrimidine-2 (1H)-thione were obtained after three days with 80% yield. Anal.Calcd for C_13_ H_15_ C_l_ N_2_ S: C, 58.53; H, 5.67; N, 10.50; S, 12.02%; found:*C*, 58.49; H,5.72; N, 10.61; S, 12.14,IR(KBr), v (cm^-1^) 1535 (C=S),1591(C=C),3184 (N—H).

### Refinement

All H atoms attached to C atoms were fixed geometrically and treated as riding with C—H= 0.93 Å(aromatic) or 0.96 Å(methy) with *U*_iso_(H)=1.2*U*_eq_(Caromatic) and 1.5U_aq_(Cmethyl). The amino hydrogen atom was located from the difference map and refined freely with *U*_iso_(H)=1.2*U*_eq_(N). In the last cycles of refinement, it was treated as riding on the parent N atom. Both methyl groups attached to C3 display rather elongated ellipsoids however no correct disordered model could be defined and these large ellipsoids may be related to dynamic motion.

### Crystal data

**Table:**

C_13_H_15_ClN_2_S	*F*(000) = 560
*M**_r_* = 266.78	*D*_x_ = 1.270 Mg m^−^^3^
Monoclinic, *P*2_1_/*c*	Melting point = 427.6–429.6 K
Hall symbol: -P 2ybc	Mo *K*α radiation, λ = 0.71073 Å
*a* = 8.398 (2) Å	Cell parameters from 2921 reflections
*b* = 14.930 (4) Å	θ = 2.2–25.5°
*c* = 11.468 (3) Å	µ = 0.40 mm^−^^1^
β = 103.909 (4)°	*T* = 298 K
*V* = 1395.7 (6) Å^3^	Block, colourless
*Z* = 4	0.40 × 0.19 × 0.17 mm

### Data collection

**Table:**

Bruker SMART APEX CCD area-detector diffractometer	2598 independent reflections
Radiation source: fine-focus sealed tube	2212 reflections with *I* > 2/s(*I*)
graphite	*R*_int_ = 0.021
Detector resolution: 83.66 pixels mm^-1^	θ_max_ = 25.5°, θ_min_ = 2.2°
ω scans	*h* = −10→10
Absorption correction: multi-scan (*SADABS*; Bruker, 2000)	*k* = −13→18
*T*_min_ = 0.855, *T*_max_ = 0.934	*l* = −12→13
8215 measured reflections	

### Refinement

**Table:**

Refinement on *F*^2^	Primary atom site location: structure-invariant direct methods
Least-squares matrix: full	Secondary atom site location: difference Fourier map
*R*[*F*^2^ > 2σ(*F*^2^)] = 0.048	Hydrogen site location: inferred from neighbouring sites
*wR*(*F*^2^) = 0.133	H-atom parameters constrained
*S* = 1.10	*w* = 1/[σ^2^(*F*_o_^2^) + (0.0696*P*)^2^ + 0.4092*P*] where *P* = (*F*_o_^2^ + 2*F*_c_^2^)/3
2598 reflections	(Δ/σ)_max_ < 0.001
157 parameters	Δρ_max_ = 0.29 e Å^−^^3^
0 restraints	Δρ_min_ = −0.14 e Å^−^^3^

### Special details

**Table:**

Geometry. All esds (except the esd in the dihedral angle between two l.s. planes) are estimated using the full covariance matrix. The cell esds are taken into account individually in the estimation of esds in distances, angles and torsion angles; correlations between esds in cell parameters are only used when they are defined by crystal symmetry. An approximate (isotropic) treatment of cell esds is used for estimating esds involving l.s. planes.
Refinement. Refinement of *F*^2^ against ALL reflections. The weighted *R*-factor *wR* and goodness of fit *S* are based on *F*^2^, conventional *R*-factors *R* are based on *F*, with *F* set to zero for negative *F*^2^. The threshold expression of *F*^2^ > σ(*F*^2^) is used only for calculating *R*-factors(gt) *etc*. and is not relevant to the choice of reflections for refinement. *R*-factors based on *F*^2^ are statistically about twice as large as those based on *F*, and *R*- factors based on ALL data will be even larger.

### Fractional atomic coordinates and isotropic or equivalent isotropic displacement parameters (Å^2^)

**Table:**

	*x*	*y*	*z*	*U*_iso_*/*U*_eq_	
Cl1	0.26790 (8)	0.97009 (5)	−0.03961 (6)	0.0647 (2)	
S1	0.77220 (7)	1.01839 (4)	0.36163 (5)	0.0496 (2)	
N1	0.8934 (2)	0.87127 (13)	0.47479 (18)	0.0508 (5)	
H1A	0.9700	0.9086	0.5030	0.061*	
N2	0.6306 (2)	0.85790 (12)	0.35950 (17)	0.0467 (5)	
C1	0.7657 (3)	0.90982 (15)	0.40192 (19)	0.0408 (5)	
C2	0.6333 (3)	0.76397 (16)	0.3814 (3)	0.0598 (7)	
C3	0.7636 (4)	0.72845 (18)	0.4552 (3)	0.0693 (8)	
H3	0.7690	0.6664	0.4621	0.083*	
C4	0.9012 (3)	0.78148 (16)	0.5276 (2)	0.0602 (7)	
C5	0.4926 (4)	0.7110 (2)	0.3124 (4)	0.1029 (13)	
H5A	0.5133	0.6484	0.3283	0.154*	
H5B	0.4787	0.7222	0.2281	0.154*	
H5C	0.3947	0.7281	0.3360	0.154*	
C7	1.0658 (5)	0.7413 (2)	0.5222 (5)	0.1179 (16)	
H7A	1.0740	0.7392	0.4401	0.177*	
H7B	1.0745	0.6818	0.5547	0.177*	
H7C	1.1528	0.7777	0.5682	0.177*	
C8	0.4808 (3)	0.90016 (15)	0.2952 (2)	0.0450 (5)	
C9	0.4507 (3)	0.91237 (15)	0.1734 (2)	0.0448 (5)	
H9	0.5261	0.8933	0.1311	0.054*	
C10	0.3064 (3)	0.95355 (15)	0.1145 (2)	0.0468 (5)	
C11	0.1924 (3)	0.98094 (17)	0.1755 (3)	0.0571 (7)	
H11	0.0959	1.0088	0.1351	0.069*	
C12	0.2238 (3)	0.96636 (19)	0.2971 (3)	0.0644 (7)	
H12	0.1467	0.9837	0.3389	0.077*	
C13	0.3679 (3)	0.92637 (18)	0.3583 (2)	0.0578 (6)	
H13	0.3887	0.9173	0.4408	0.069*	
C6	0.8890 (7)	0.7904 (3)	0.6570 (3)	0.137 (2)	
H6A	0.9756	0.8284	0.7001	0.206*	
H6B	0.8989	0.7323	0.6940	0.206*	
H6C	0.7849	0.8161	0.6590	0.206*	

### Atomic displacement parameters (Å^2^)

**Table:**

	*U*^11^	*U*^22^	*U*^33^	*U*^12^	*U*^13^	*U*^23^
Cl1	0.0586 (4)	0.0726 (5)	0.0561 (4)	−0.0055 (3)	0.0005 (3)	0.0109 (3)
S1	0.0455 (4)	0.0420 (3)	0.0541 (4)	−0.0027 (2)	−0.0022 (3)	0.0110 (3)
N1	0.0452 (11)	0.0431 (11)	0.0568 (11)	0.0034 (8)	−0.0022 (9)	0.0078 (9)
N2	0.0430 (10)	0.0391 (10)	0.0546 (11)	−0.0009 (8)	0.0050 (8)	0.0046 (8)
C1	0.0399 (11)	0.0419 (12)	0.0397 (11)	0.0035 (9)	0.0077 (9)	0.0014 (9)
C2	0.0618 (16)	0.0402 (13)	0.0743 (17)	−0.0030 (11)	0.0104 (13)	0.0033 (12)
C3	0.082 (2)	0.0373 (13)	0.081 (2)	0.0015 (13)	0.0059 (16)	0.0088 (13)
C4	0.0760 (18)	0.0425 (13)	0.0546 (14)	0.0126 (12)	0.0010 (12)	0.0076 (11)
C5	0.082 (2)	0.0519 (17)	0.158 (4)	−0.0206 (16)	−0.004 (2)	0.008 (2)
C7	0.080 (2)	0.064 (2)	0.185 (5)	0.0305 (18)	−0.015 (2)	−0.003 (3)
C8	0.0378 (11)	0.0412 (12)	0.0534 (13)	−0.0050 (9)	0.0056 (9)	−0.0017 (10)
C9	0.0393 (11)	0.0438 (12)	0.0520 (13)	−0.0049 (9)	0.0123 (10)	−0.0022 (10)
C10	0.0412 (12)	0.0414 (11)	0.0533 (13)	−0.0084 (10)	0.0025 (10)	0.0012 (10)
C11	0.0412 (13)	0.0535 (15)	0.0714 (17)	0.0033 (11)	0.0035 (12)	−0.0057 (12)
C12	0.0506 (15)	0.0716 (19)	0.0732 (19)	0.0066 (12)	0.0195 (14)	−0.0122 (14)
C13	0.0538 (15)	0.0655 (17)	0.0546 (14)	0.0006 (12)	0.0142 (12)	−0.0055 (12)
C6	0.270 (6)	0.082 (3)	0.058 (2)	−0.033 (3)	0.035 (3)	0.0098 (19)

### Geometric parameters (Å, °)

**Table:**

Cl1—C10	1.736 (3)	C7—H7A	0.9600
S1—C1	1.690 (2)	C7—H7B	0.9600
N1—C1	1.323 (3)	C7—H7C	0.9600
N1—C4	1.466 (3)	C8—C9	1.372 (3)
N1—H1A	0.8541	C8—C13	1.380 (3)
N2—C1	1.364 (3)	C9—C10	1.382 (3)
N2—C2	1.424 (3)	C9—H9	0.9300
N2—C8	1.441 (3)	C10—C11	1.376 (4)
C2—C3	1.323 (4)	C11—C12	1.372 (4)
C2—C5	1.483 (4)	C11—H11	0.9300
C3—C4	1.480 (4)	C12—C13	1.381 (4)
C3—H3	0.9300	C12—H12	0.9300
C4—C6	1.518 (5)	C13—H13	0.9300
C4—C7	1.522 (4)	C6—H6A	0.9600
C5—H5A	0.9600	C6—H6B	0.9600
C5—H5B	0.9600	C6—H6C	0.9600
C5—H5C	0.9600		
			
C1—N1—C4	127.4 (2)	H7A—C7—H7B	109.5
C1—N1—H1A	112.2	C4—C7—H7C	109.5
C4—N1—H1A	119.1	H7A—C7—H7C	109.5
C1—N2—C2	121.35 (19)	H7B—C7—H7C	109.5
C1—N2—C8	118.85 (18)	C9—C8—C13	121.0 (2)
C2—N2—C8	119.75 (18)	C9—C8—N2	120.2 (2)
N1—C1—N2	117.2 (2)	C13—C8—N2	118.8 (2)
N1—C1—S1	121.01 (17)	C8—C9—C10	118.8 (2)
N2—C1—S1	121.78 (15)	C8—C9—H9	120.6
C3—C2—N2	118.9 (2)	C10—C9—H9	120.6
C3—C2—C5	123.9 (2)	C11—C10—C9	121.3 (2)
N2—C2—C5	117.0 (2)	C11—C10—Cl1	119.56 (19)
C2—C3—C4	124.0 (2)	C9—C10—Cl1	119.16 (19)
C2—C3—H3	118.0	C12—C11—C10	118.8 (2)
C4—C3—H3	118.0	C12—C11—H11	120.6
N1—C4—C3	107.8 (2)	C10—C11—H11	120.6
N1—C4—C6	108.5 (2)	C11—C12—C13	121.0 (2)
C3—C4—C6	111.6 (3)	C11—C12—H12	119.5
N1—C4—C7	107.1 (3)	C13—C12—H12	119.5
C3—C4—C7	111.2 (3)	C8—C13—C12	119.0 (2)
C6—C4—C7	110.5 (3)	C8—C13—H13	120.5
C2—C5—H5A	109.5	C12—C13—H13	120.5
C2—C5—H5B	109.5	C4—C6—H6A	109.5
H5A—C5—H5B	109.5	C4—C6—H6B	109.5
C2—C5—H5C	109.5	H6A—C6—H6B	109.5
H5A—C5—H5C	109.5	C4—C6—H6C	109.5
H5B—C5—H5C	109.5	H6A—C6—H6C	109.5
C4—C7—H7A	109.5	H6B—C6—H6C	109.5
C4—C7—H7B	109.5		
			
C4—N1—C1—N2	−8.0 (4)	C2—C3—C4—C6	100.9 (4)
C4—N1—C1—S1	173.1 (2)	C2—C3—C4—C7	−135.3 (3)
C2—N2—C1—N1	−7.0 (3)	C1—N2—C8—C9	88.4 (3)
C8—N2—C1—N1	170.6 (2)	C2—N2—C8—C9	−94.0 (3)
C2—N2—C1—S1	171.87 (19)	C1—N2—C8—C13	−92.8 (3)
C8—N2—C1—S1	−10.5 (3)	C2—N2—C8—C13	84.9 (3)
C1—N2—C2—C3	7.4 (4)	C13—C8—C9—C10	1.5 (3)
C8—N2—C2—C3	−170.1 (3)	N2—C8—C9—C10	−179.65 (19)
C1—N2—C2—C5	−168.4 (3)	C8—C9—C10—C11	−1.0 (3)
C8—N2—C2—C5	14.1 (4)	C8—C9—C10—Cl1	179.51 (17)
N2—C2—C3—C4	6.8 (4)	C9—C10—C11—C12	−0.3 (4)
C5—C2—C3—C4	−177.7 (3)	Cl1—C10—C11—C12	179.2 (2)
C1—N1—C4—C3	19.4 (4)	C10—C11—C12—C13	1.1 (4)
C1—N1—C4—C6	−101.6 (3)	C9—C8—C13—C12	−0.7 (4)
C1—N1—C4—C7	139.1 (3)	N2—C8—C13—C12	−179.6 (2)
C2—C3—C4—N1	−18.2 (4)	C11—C12—C13—C8	−0.6 (4)

### Hydrogen-bond geometry (Å, °)

**Table:**

*D*—H···*A*	*D*—H	H···*A*	*D*···*A*	*D*—H···*A*
N1—H1A···S1^i^	0.85	2.58	3.404 (2)	162

Symmetry codes: (i) −*x*+2, −*y*+2, −*z*+1.
